# Endogenous μ-opioid—Neuropeptide Y Y1 receptor synergy silences chronic postoperative pain in mice

**DOI:** 10.1093/pnasnexus/pgad261

**Published:** 2023-08-14

**Authors:** Tyler S Nelson, Diogo F S Santos, Pranav Prasoon, Margaret Gralinski, Heather N Allen, Bradley K Taylor

**Affiliations:** Department of Anesthesiology and Perioperative Medicine, Center for Neuroscience, Pittsburgh Center for Pain Research, Pittsburgh Project to end Opioid Misuse, University of Pittsburgh School of Medicine, 200 Lothrop Street, Pittsburgh, PA 15213, USA; Center for the Neural Basis of Cognition, University of Pittsburgh, Pittsburgh, PA 15213, USA; Department of Anesthesiology and Perioperative Medicine, Center for Neuroscience, Pittsburgh Center for Pain Research, Pittsburgh Project to end Opioid Misuse, University of Pittsburgh School of Medicine, 200 Lothrop Street, Pittsburgh, PA 15213, USA; Department of Anesthesiology and Perioperative Medicine, Center for Neuroscience, Pittsburgh Center for Pain Research, Pittsburgh Project to end Opioid Misuse, University of Pittsburgh School of Medicine, 200 Lothrop Street, Pittsburgh, PA 15213, USA; Department of Anesthesiology and Perioperative Medicine, Center for Neuroscience, Pittsburgh Center for Pain Research, Pittsburgh Project to end Opioid Misuse, University of Pittsburgh School of Medicine, 200 Lothrop Street, Pittsburgh, PA 15213, USA; Department of Anesthesiology and Perioperative Medicine, Center for Neuroscience, Pittsburgh Center for Pain Research, Pittsburgh Project to end Opioid Misuse, University of Pittsburgh School of Medicine, 200 Lothrop Street, Pittsburgh, PA 15213, USA; Department of Anesthesiology and Perioperative Medicine, Center for Neuroscience, Pittsburgh Center for Pain Research, Pittsburgh Project to end Opioid Misuse, University of Pittsburgh School of Medicine, 200 Lothrop Street, Pittsburgh, PA 15213, USA

**Keywords:** latent sensitization, μ-opioid receptor, neuropeptide Y Y1 receptor, spinal cord dorsal horn, synergy, G-protein-coupled receptor, endogenous analgesia, chronic postsurgical pain

## Abstract

Tissue injury creates a delicate balance between latent pain sensitization (LS) and compensatory endogenous analgesia. Inhibitory G-protein-coupled receptor (GPCR) interactions that oppose LS, including μ-opioid receptor (MOR) or neuropeptide Y Y1 receptor (Y1R) activity, persist in the spinal cord dorsal horn (DH) for months, even after the resolution of normal pain thresholds. Here, we demonstrate that following recovery from surgical incision, a potent endogenous analgesic synergy between MOR and Y1R activity persists within DH interneurons to reduce the intensity and duration of latent postoperative hypersensitivity and ongoing pain. Failure of such endogenous GPCR signaling to maintain LS in remission may underlie the transition from acute to chronic pain states.

**Significance Statement**

Chronic postsurgical pain occurs in up to 50% of patients following surgery and produces enormous emotional, physical, and economic costs. Unfortunately, the complex physiological mechanisms that lead to the manifestation of chronic postsurgical pain remain unknown and efficacious treatments do not exist. Here, in a preclinical latent sensitization (LS) model of chronic postsurgical pain, we reveal that two spinal inhibitory G-protein-coupled receptors (GPCRs), μ-opioid receptors, and neuropeptide Y Y1 receptors, synergistically maintain chronic postsurgical pain in remission, long after the resolution of normal pain thresholds. Failure of this endogenous GPCR signaling to maintain LS in remission may underlie the transition from acute to chronic pain states.

## Introduction

Chronic postsurgical pain (CPSP) is a significant healthcare burden that afflicts millions of patients each year (1, 2). Despite this high prevalence, the biological mechanisms that underlie the transition from acute pain to CPSP remain poorly understood (3, 4). The dorsal horn (DH) of the spinal cord processes somatosensory information and is a key driver of pathological pain states (5). Tissue injury sensitizes pro-nociceptive neurons in the DH, contributing to allodynia and hyperalgesia (6–8). However, accumulating evidence from human and animal studies suggest that after tissue-injury-induced hyperalgesia resolves, sensitization in the DH persists within a long-lasting silent state of remission, termed “latent sensitization” (LS) (9, 10).

Following tissue injury and the subsequent resolution of hyperalgesia, intrathecal (i.t.) administration of selective antagonists at inhibitory Gα_i/o_ G-protein-coupled receptors (GPCRs), including μ-opioid receptors (MORs), κ-opioid receptors (KORs), neuropeptide Y Y1 receptors (Y1Rs), or several other receptors, unmask LS and reinstate hyperalgesia (11–16). Remarkably, each antagonist was sufficient to produce a complete, not partial, reinstatement of hyperalgesia. This suggested that GPCRs interact in a complex manner, not just additively, to maintain LS in remission. Indeed, individual cells express many GPCRs whose intracellular second messengers can interact to coalter signaling (17–20). For example, different GPCRs can activate the same G proteins (21, 22). Thus, coincidental activation of second messenger pathways by coactivation of multiple GPCRs can elicit supra-additive (synergistic) amplification of the responses and produce a greater than additive leftward shift in the response curve (23–26).

Examples of spinal analgesic synergy between Gα_i/o_ GPCR agonists exist in the pharmacology literature, including μ- and κ-selective opiates, or μ- and δ-selective opiates (27, 28), opiates and cannabinoids (29–31), and opiates and α2-adrenergic receptor agonists (32–34). The aim of this study wasto test the hypothesis that surgical incision produces a tonic and long-lasting synergistic dependence on MOR and Y1R endogenous signaling to oppose CPSP.

## Results

### MOR and Y1R are coexpressed in dorsal root ganglion and DH

**Fig. 1.**
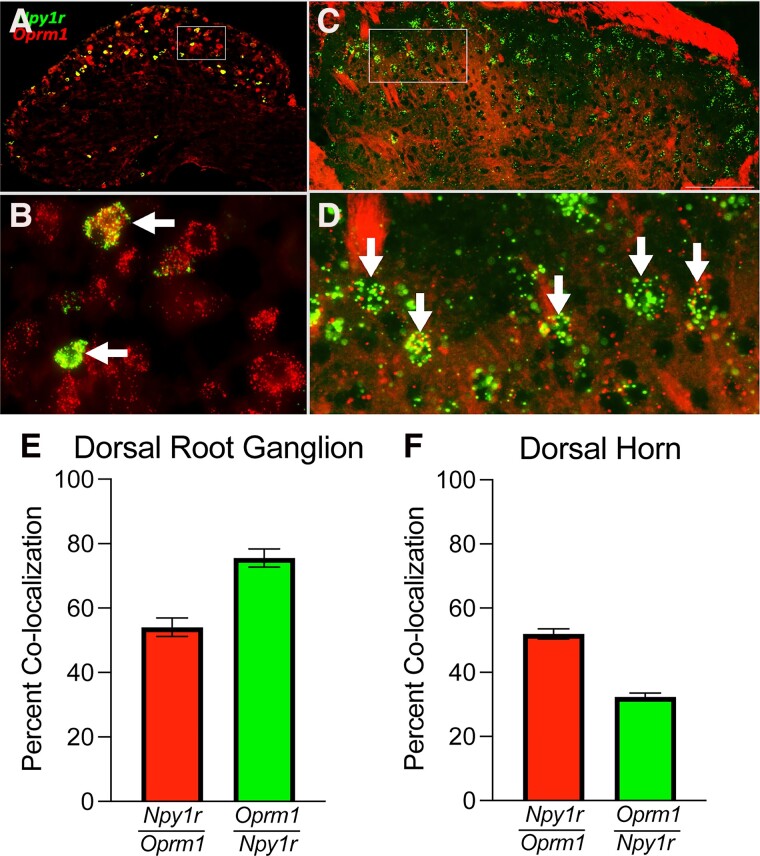
Y1R and MOR are coexpressed in the DRG and DH. A–D) FISH demonstrating colocalization of *Oprm1* and *Npy1r* in the same cells in both lumbar DRG A and B) and DH C and D). B and D) represent zoomed in box insets for A and C, respectively. Arrows indicate colocalization. E) *Npy1r* colocalizes with *Oprm1* in the DRG (*Npy1r*/*Oprm1*—54.08 ± 2.85%; *Oprm1*/*Npy1r*—75.54 ± 2.87%). F) *Npy1r* colocalizes with *Oprm1* in the spinal cord DH (*Npy1r*/*Oprm1*—51.96 ± 1.59%; *Oprm1*/*Npy1r*—32.28 ± 1.22%). Averages are determined from 4 to 5 quantified sections/mouse, *n* = 3 mice/group. Data are shown as mean ± SEM.

Synergistic interactions between MOR and Y1R may be mediated by either (i) intracellular mechanisms in which receptors located on the same cell produce interactions at the level of intracellular signaling cascades, or (ii) via intercellular mechanisms that involve coincident inhibition of two neurons in series in the same anatomical pathway or a retrograde feedback mechanism (32). First, we examined MOR and Y1R localization using fluorescence in situ hybridization (FISH) for *Oprm1* and *Npy1r*. We found cellular colocalization in the lumbar dorsal root ganglion (DRG; Fig. 1A, B, E) and DH (Fig. 1C, D, F). Thus, MOR and Y1R intracellular neuronal cross-talk in both DRG and DH is plausible to produce synergistic intracellular signaling.

### MOR and Y1R signaling work synergistically to oppose CPSP

**Fig. 2.**
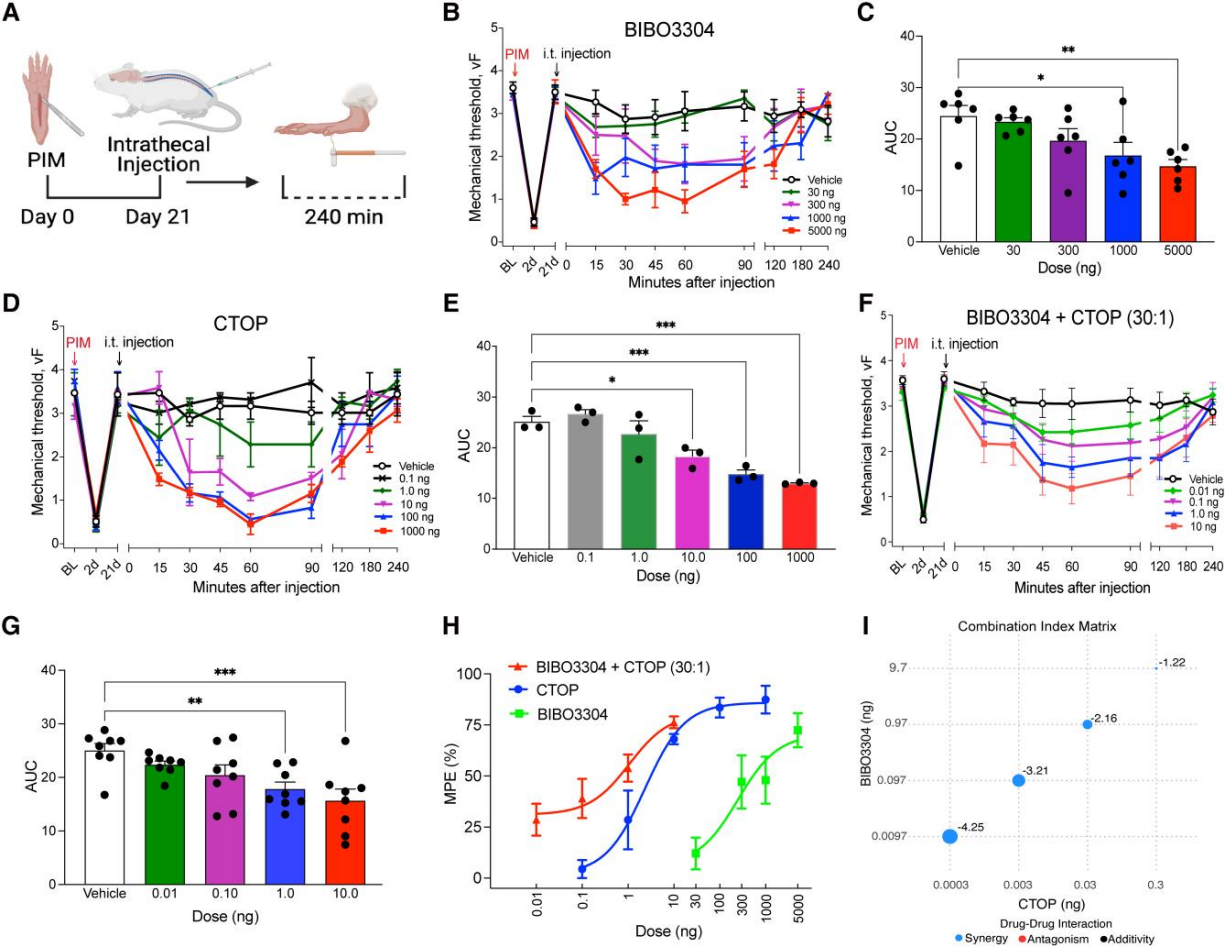
Y1R and MOR synergistically oppose LS. A) Experimental timeline of PIM, i.t. injections into the mouse to pharmacologically target the spinal cord, and von Frey mechanical threshold behavioral testing. B) Dose–response time course of reinstatement of hyperalgesia after i.t. administration of BIBO3304 and C) area under the curve analysis. *n* = 6 mice/group. One-way ANOVA: *F*(4, 25) = 4.694, *P* = 0.0058; Dunnett's multiple comparison tests. D) Dose–response time course of reinstatement of hyperalgesia after i.t. administration of CTOP and E) area under the curve analysis. *n* = 3 mice/group. One-way ANOVA: *F*(5, 12) = 17.09, *P* < 0.0001; Dunnett's multiple comparison tests. G) Dose–response time course of reinstatement of hyperalgesia after i.t. administration of BIBO3304 and CTOP in combination and H) area under the curve analysis (*n* = 8 mice/group). One-way ANOVA: *F*(4, 35) = 5.510, *P* = 0.0015; Dunnett's multiple comparison tests. H) Dose–response effects of antagonist-induced reinstatement (MPE). I) CI matrix showing Log CI values for MPE from mice treated with the drug combination of BIBO3304 and CTOP. The larger the size of the circle is the higher the CI power. Analyses were performed using SiCoDEA software. Data in B–H are shown as mean ± SEM. *P < 0.05, **P < 0.01, and ***P < 0.001.

Next, we performed a plantar incision model (PIM) of postoperative pain that produces robust mechanical hyperalgesia that resolves within 21 days (Fig. 2A). Following the resolution of hyperalgesia, we performed i.t. administration of a MOR antagonist (CTOP) or an Y1R antagonist (BIBO3304). Intrathecal administration delivers the injectate to the subarachnoid space (below the dura mater and arachnoid mater) and bypasses the blood–brain barrier. BIBO3304 and CTOP dose-dependently reinstated mechanical hypersensitivity (Fig. 2B–E). Preliminary experimentation suggested that BIBO3304 exhibited a 30-fold difference in potency compared with CTOP; therefore, we next assessed synergistic interactions with a fixed ratio (30:1) isobologram method (35, 36). BIBO3304 and CTOP combination (BIBO3304:CTOP) reinstated mechanical hypersensitivity with robust effects at even a remarkably low 100 pg dose (Fig. 2F–G). BIBO3304:CTOP produced a large leftward shift in the dose–response curve when compared with either BIBO3304 or CTOP administered alone (Fig. 2H), and the combinatorial drug interaction analysis revealed a significant synergistic interaction for all dose combinations (Fig. 2I).

**Fig. 3.**
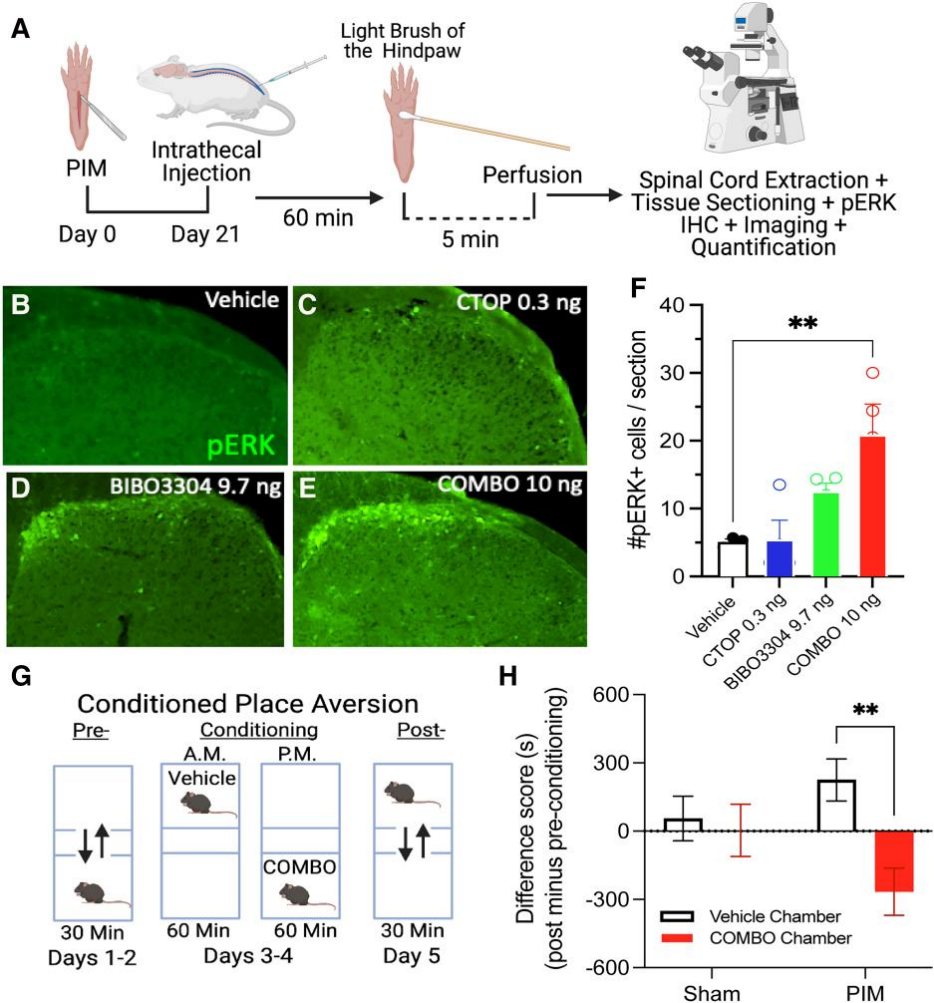
Synergistic inhibition of Y1R and MOR is sufficient to induce immunohistochemical and behavioral markers of sensory and affective pain. A) Experimental timeline for PIM, i.t. pharmacology, light brush of the hind paw, and immunohistochemical staining for pERK immunoreactivity in the lumbar DH. B–E) Representative images of ipsilateral DH light-touched evoked pERK immunoreactivity after i.t. administration of agents. F) Intrathecally administered BIB03304:CTOP (10.0 ng) induces light-touched evoked pERK immunoreactivity in the ipsilateral DH. *n* = 3–4 mice/group. One-way ANOVA: *F*(3, 11) = 6.749, *P* = 0.0076; Dunnett's multiple comparison tests. G) Experimental protocol for CPA. H) BIB03304:CTOP (10.0 ng, i.t.) induces CPA in postincision but not postsham incision mice. *n* = 11–12 mice/group. Two-way ANOVA: interaction *F*(1, 42) = 4.606, *P* = 0.0377. Holm–Sidak post hoc test. Data are presented as mean ± SEM.

In addition to mechanical hypersensitivity (reduction of von Frey withdrawal threshold), BIBO3304:CTOP (10 ng, i.t.) also dramatically increased the light touch-evoked expression of phosphorylated extracellular signal-regulated kinase (pERK) in superficial DH neurons, a proxy for neuronal activation (Fig. 3A–F). Additionally, we tested the hypothesis that BIBO3304:CTOP (10 ng, i.t.) is sufficient to produce avoidance using a conditioned place aversion (CPA) paradigm (Fig. 3G). BIBO3304:CTOP (10 ng, i.t.) produced a robust CPA in mice with plantar but not sham incision (Fig. 3H). Together, these results demonstrate that Y1R and MOR synergistically oppose incision-induced LS to maintain in remission both the sensory and affective dimensions of chronic pain.

### MOR and Y1R signaling within DH rather than DRG neurons synergistically opposes a GluN2A *N*-methyl-d-aspartate receptor–dependent LS

**Fig. 4.**
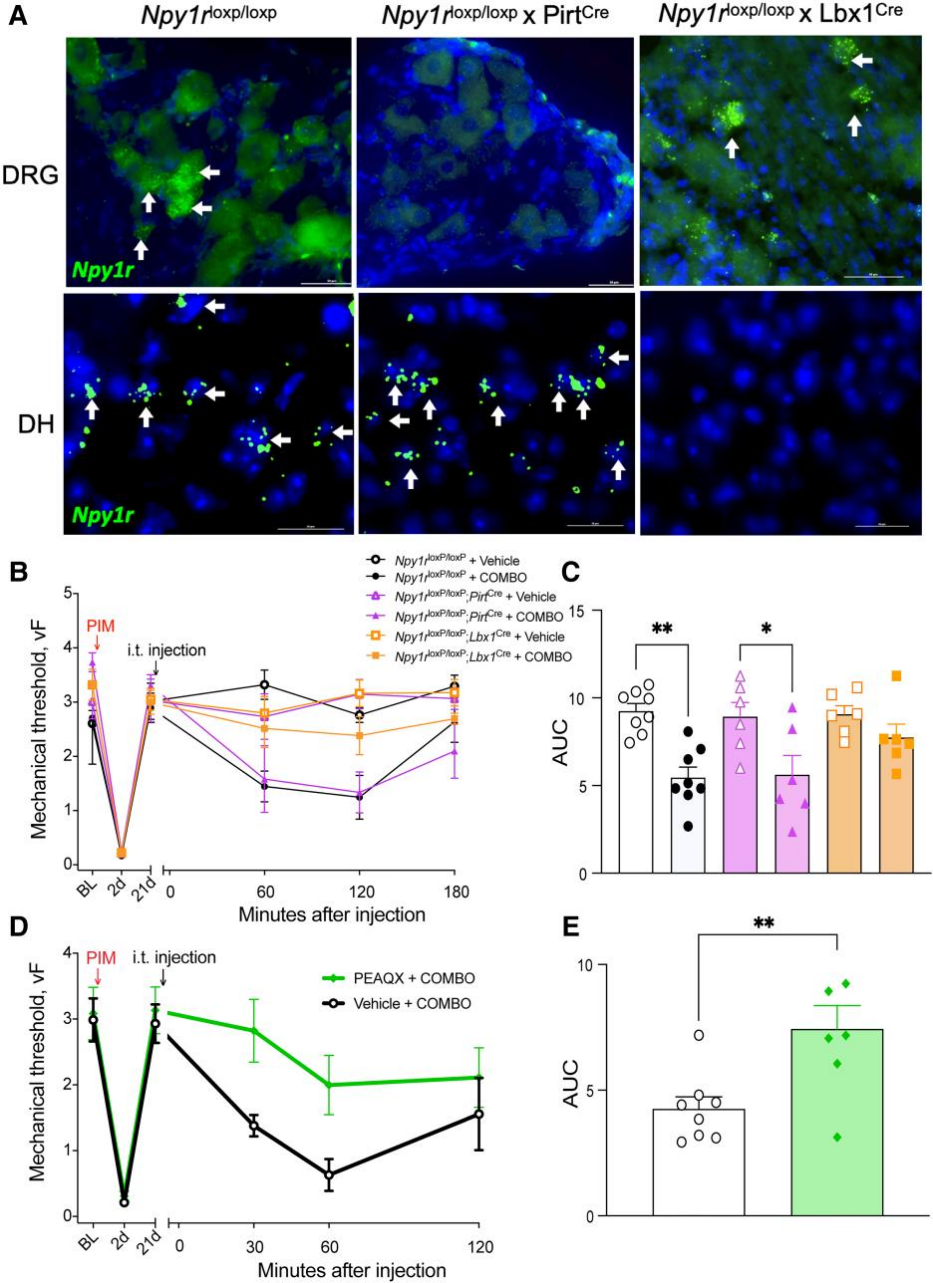
DH but not DRG Y1R and MOR synergy opposes a GluN2-driven LS. A) FISH of sections through DRG (top row) and DH of the spinal cord (bottom row) demonstrate that *Npy1r*^loxP/loxP^ mice express *Npy1r* in DRG and spinal cord, *Npy1r*^loxP/loxP^; *Pirt*^Cre^ mice lack expression of *Npy1r* in the DRG, and *Npy1r*^loxP/loxP^; *Lbx1*^Cre^ mice lack expression of *Npy1r* in the DH of the spinal cord. B) Dose–response time course of reinstatement of incision-induced mechanical hyperalgesia after administration of BIBO3304:CTOP (10.0 ng, i.t.) in *Npy1r*^loxP/loxP^ and *Npy1r*^loxP/loxP^; *Pirt*^Cre^ mice but not in Npy1r^loxP/loxP^; *Lbx1*^Cre^ mice and C) area under the curve analysis. *n* = 6–9 mice/group. One-way ANOVA: *F*(5, 34) = 6.442, *P* = 0.0003; Tukey's multiple comparison tests. D and E) A GluN2a NMDAR subtype antagonist, PEAQX (100 ng, i.t.), prevented BIBO3304:CTOP-induced reinstatement of mechanical hyperalgesia (D) dose–response curve and area under the curve (E). *n* = 7–8 mice/group. Unpaired two-tailed *T* test, *P* = 0.0073. Data are presented as mean ± SEM. *P < 0.05 and **P < 0.01.

Intrathecally administered chemicals can engage both DH and DRG neurons. To resolve the specific site of action for BIBO3304:CTOP, we used a conditional genetic knockout approach combined with pharmacology. Specifically, we crossed *Npy1r*^loxP/loxP^ mice with either *Pirt*^Cre^ or *Lbx1*^Cre^ mice to conditionally knockout *Npy1r* in the DRG or DH (37), respectively (Fig. 4A). *Npy1r^l^*^oxP/loxP^; *Pirt*^Cre^ and *Npy1r*^loxP/loxP^; *Lbx1*^Cre^ mice developed incision-induced hypersensitivity that resolved within 21 days; therefore, we were able to probe mechanisms of LS using these genetic crosses. Remarkably, BIBO3304:CTOP (10 ng, i.t.) reinstated incision-induced mechanical hypersensitivity in both control (*Npy1r^l^*^oxP/loxP^) and DRG conditional knockout mice (*Npy1r*^loxP/loxP^; *Pirt*^Cre^), but not in DH conditional knockout mice (*Npy1r*^loxP/loxP^; *Lbx1*^Cre^) (Fig. 4B and C). These data suggest that MOR and Y1R signal within DH neurons, rather than DRG neurons, to synergistically oppose LS and maintain postoperative pain in remission.

We next determined whether the pro-nociceptive DH neuron activity that is synergistically inhibited by MOR and Y1R is dependent on *N*-methyl-d-aspartate receptors (NMDARs). Previously, we reported that an NMDAR blocker, MK-801 (dizocilpine), prevented either Y1R (13) or MOR (12) antagonist-induced reinstatement of peripheral inflammatory pain. Therefore, we hypothesized that NMDARs, in particular the GluN2A NMDAR subunit (encoded by *Grin2a*) that is necessary for the manifestation of inflammatory pain (38) and one of the top three “hub” genes implicated in CPSP (39), also mediate the LS that is unmasked by BIBO3304:CTOP. To test this, we coadministered the GluN2A-preferring NMDAR antagonist, PEAQX (100 ng, i.t.), with BIBO3304:CTOP (10 ng, i.t.). PEAQX, but not vehicle, abolished the BIBO3304:CTOP-induced reinstatement of mechanical hypersensitivity (Fig. 4D and E). These data suggest that MOR and Y1R signaling synergistically opposes a GluN2A-mediated LS to maintain postoperative pain in remission.

### MOR and Y1R agonists inhibit postoperative pain, but not in a synergistic manner

**Fig. 5.**
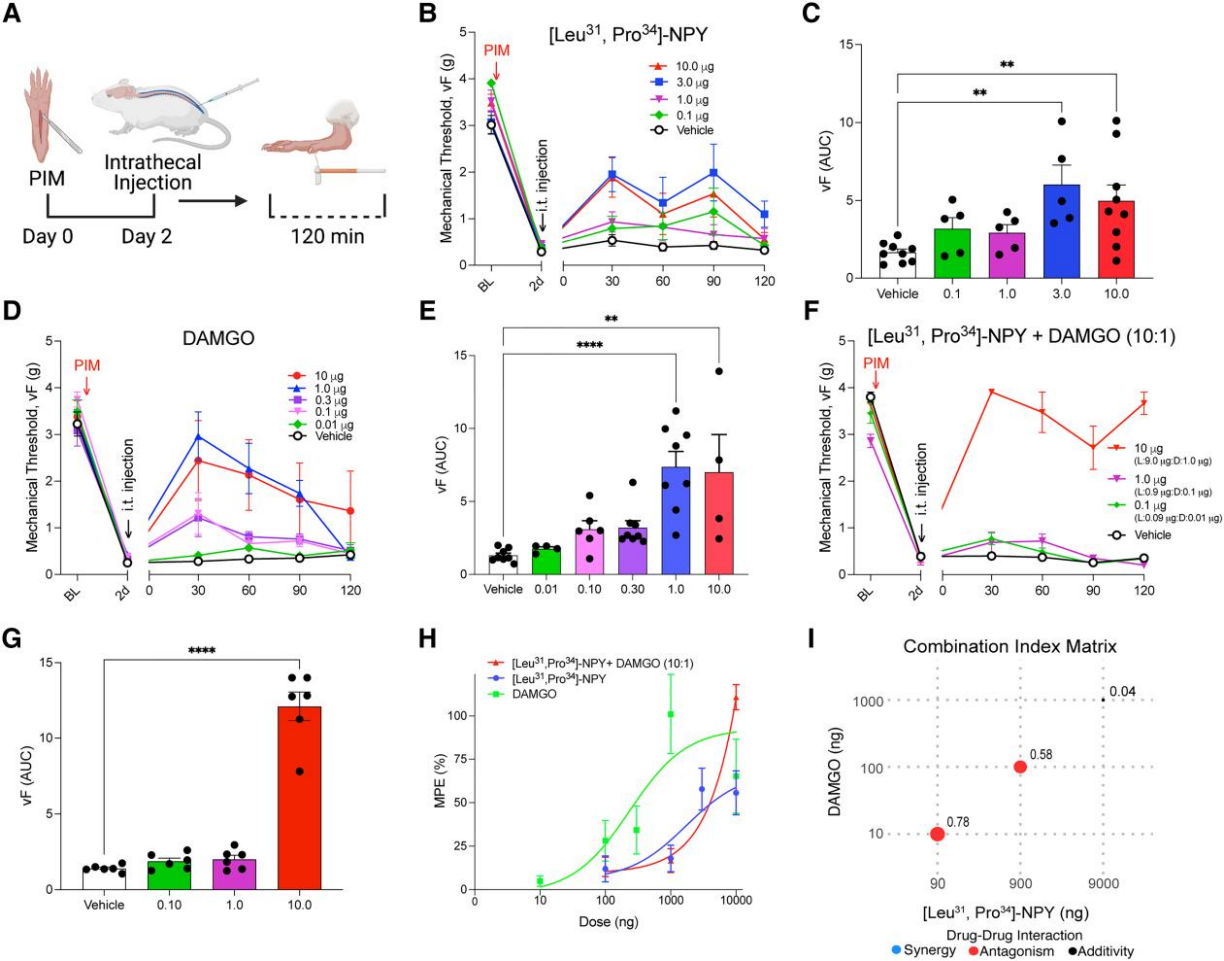
Exogenous Y1R and MOR agonists do not synergistically inhibit acute pain. A) Experimental timeline of PIM, i.t. injections (i.t.) into the mouse to pharmacologically target the spinal cord, and von Frey mechanical threshold behavioral testing. B and C) Intrathecal administration of [Leu^31^, Pro^34^]-NPY dose-dependently (0.1, 1.0, 3.0, or 10.0 µg) reduced incision-induced hyperalgesia. *n* = 5–9 mice/group. One-way ANOVA: *F*(4, 28) = 4.707, *P* = 0.0049; Dunnett's multiple comparison tests. D and E) Intrathecal administration of DAMGO dose-dependently (0.01, 0.1, 0.3, 1.0, or 10.0 µg) reduced incision-induced hyperalgesia. *n* = 4–8 mice/group. One-way ANOVA: *F*(5, 32) = 8.316, *P* < 0.0001; Dunnett's multiple comparison tests. F and G) Intrathecal administration of [Leu^31^, Pro^34^]-NPY and DAMGO together in a 10:1 combination reduces incision-induced hyperalgesia in an additive manner. *n* = 6 mice/group. One-way ANOVA: *F*(3, 20) = 103.6, *P* < 0.0001; Dunnett's multiple comparison tests. H) Dose–response effects of agonist-induced antihyperalgesia (MPE). I) CI matrix showing Log CI values for MPE from mice treated with the drug combination of [Leu^31^, Pro^34^]-NPY and DAMGO. The larger the size of the circle is the higher the CI power. Analyses were performed using SiCoDEA software. Data in B–H are shown as mean ± SEM. **P < 0.01 and ****P < 0.0001.

Next, we asked if *exogenously* administered Y1R and MOR agonists would exert antihyperalgesic effects in a synergistic manner. To do this, we delivered agonists by themselves and in combination 2 days after plantar incision. First, we tested the ability of the Y1R-selective agonist, [Leu^31^, Pro^34^]-NPY, or the MOR agonist, DAMGO, to inhibit mechanical hypersensitivity (Fig. 5A). We found that both [Leu^31^, Pro^34^]-NPY (0.1, 1.0, 3.0, or 10.0 µg, i.t.; Fig. 5B and C) and DAMGO (0.01, 0.1, 0.30, 1.0, or 10.0 µg, i.t.; (Fig. 5D and E) dose dependently reduced incision-induced mechanical hypersensitivity. Preliminary behavior studies that we performed suggested that DAMGO exhibited a 10-fold greater potency compared with [Leu^31^, Pro^34^]-NPY; therefore, we next assessed combinatorial interactions with a fixed 10:1 ratio. The [Leu^31^, Pro^34^]-NPY:DAMGO combination (10:1, i.t.) reduced mechanical hypersensitivity (Fig. 5F and G). The dose–response curve was not shifted to the left or right of [Leu^31^, Pro^34^]-NPY alone (Fig. 5H), and the combinatorial drug interaction analysis revealed no significant synergistic interaction for any dose combinations (Fig. 5I). On the contrary, the lowest dose combinations exhibited slight antagonism, while the highest dose combination exhibited an additive combinatorial effect (Fig. 5I). Together, these results demonstrate that combinatorial administration of the Y1R agonist, [Leu^31^, Pro^34^]-NPY, and the MOR agonist, DAMGO, does not inhibit acute PIM-induced hyperalgesia in a supra-additive (synergistic) manner.

## Discussion

We employed combinatorial drug interaction analyses to examine both endogenous and exogenous Y1R and MOR synergy. To assess drug interaction, the observed response of a drug combination is compared with the expected effect assuming no interaction, as determined by a reference model. Synergistic combinations are identified when the response exceeds expectations, while antagonism is determined when the response falls below expectations. The two most stringent and widely used reference models are the Loewe additivity model (40) and the Bliss independence model (41). These models, along with their variants and extensions, have been developed based on different assumptions regarding the expected noninteraction effect. The Loewe additivity model assumes that the expected effect of a drug combination is equivalent to combining the drug with itself. In contrast, the Bliss independence model uses probabilistic theory to treat the effects of individual drugs in a combination as independent and competing events. However, the differing assumptions of these models have led to a lack of agreement on which reference model should be used in an unbiased and statistically robust manner (42, 43). In this study, we employed the recently developed zero interaction potency (ZIP) model which has emerged as an alternative and hybrid approach in synergy analysis that combines the advantages of both the Loewe and Bliss models (44). Furthermore, in contrast to the Bliss and Loewe models, the ZIP model does not assume any specific type of interaction between drugs and allows for an unbiased assessment of drug combinations without imposing preconceived notions of interaction. This lack of assumption is particularly advantageous in situations where there is limited knowledge or uncertainty about the nature of drug interactions. An additional advantage of the ZIP model is that, rather than relying on a single parameter to assess drug interaction, it provides an interaction landscape over the full dose–response matrix to identify and quantify synergistic and antagonistic dose regions. Using the ZIP model, we discovered endogenous analgesic synergy between MOR and Y1R signaling that persists beyond the resolution of hyperalgesia and injury. If this maintains CPSP in remission, then a modest failure in either Y1R or MOR compensatory signaling could unmask vulnerability to remission, leading to the presentation of CPSP.

**Fig. 6.**
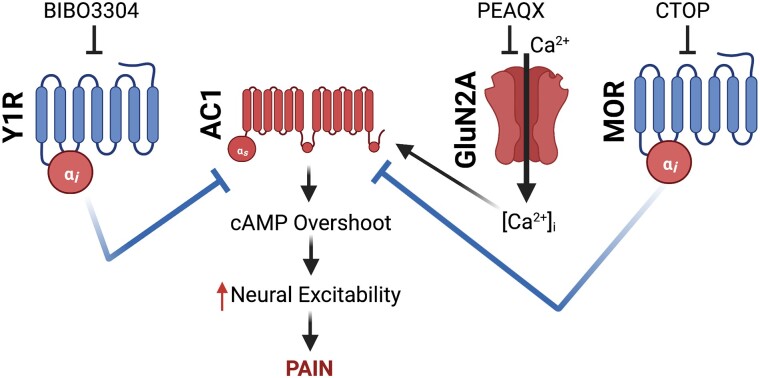
Proposed schematic of cellular pathways involved in endogenous NPY and opioid synergistic pain inhibition. We propose that following initial pain resolution and the accompanying development of LS, endogenous ligand-dependent (NPY binding to Y1R) and ligand-independent (μ-opioid receptor constitutive activity, MOR_CA_) interact in a synergistic manner to maintain LS in remission. However, this long-lasting Gα_i/o_-coupled GPCR activity produces heterologous sensitization of AC1. We hypothesize that both MOR and Y1R share a common pool of AC1, thus, potent activation or blockade of either MOR_CA_ or Y1R signaling can prevent or produce a cAMP overshoot and the reinstatement of hyperalgesia, respectively. This idea is an extension of the work of Levitt et al. (52).

Cells must integrate multiple signals from an array of receptors at any given moment. One of the most fundamental and evolutionarily conserved signaling mechanisms is GPCR activation, which is classically viewed as a compartmentalized cellular event in which a ligand binds a receptor to activate a specific signaling pathway distinct from other GPCRs (20, 45, 46). However, researchers are uncovering examples of how GPCRs and their intracellular second messengers might interact within a cell to supra-additively coalter signaling (17–20, 26, 28, 34). MOR and Y1R are *Pertussis* toxin-sensitive Gα_i/o_-coupled GPCRs; thus, upon initial receptor activation, the Gα_i/o_ subunit potently inhibits adenylyl cyclase to reduce the production of cAMP. The free Gβγ counterpart acts as a signaling molecule to activate downstream signaling pathways that include activation of G-protein-coupled inwardly rectifying K^+^ channels and inhibition of voltage-gated Ca^2+^ channels to reduce the excitability of neurons (47). Paradoxically, prolonged activation of Gα_i/o_ GPCRs enhances the activity of adenylyl cyclase and markedly increases cAMP production. This cellular phenomenon is referred to as heterologous sensitization (otherwise referred to as supersensitization, cAMP overshoot, cAMP superactivation) and is readily apparent upon removal of the agonist (48–50). Interestingly, blockade of MOR constitutive activity in the setting of LS produces heterologous sensitization of adenylyl cyclase 1 (AC1) (12). This likely occurs for spinal Y1Rs as well, as the endogenous ligand, NPY, also produces heterologous sensitization (51), and Y1R antagonism-induced reinstatement of pain-like behavior is lost in AC1 knockout mice (13, 14). Our current results suggest that endogenous antinociceptive peptides (e.g. enkephalins, endorphine, and NPY) interact with MOR and Y1R in a synergistic manner to maintain LS in remission. Gα_i/o_-coupled GPCRs share a common pool of adenylyl cyclase, thus, when one Gα_i/o_-coupled GPCR produces heterologous sensitization, administration of a different Gα_i/o_ GPCR agonist can prevent subsequent cAMP overshoot (52). As schematized in Fig. 6, we suggest that endogenous MOR and Y1R activity synergistically inhibit AC1 while counter-adaptively producing a heterologous sensitization of AC1. Antagonism of the synergistically interacting, LS-inhibiting, Gα_i/o_-coupled GPCRs is therefore sufficient to evoke a cAMP overshoot and unmask LS to produce a complete reinstatement of hyperalgesia.

We were unable to demonstrate analgesic drug synergy when tested with MOR- and Y1R-selective agonists. However, our studies used the very potent MOR agonist, DAMGO (53), and so floor effects may have masked the interactions with [Leu^31^, Pro^34^]-NPY. To address this caveat, future studies could assess the interactions of MOR agonists with very different properties, such as beta-endorphin, endomorphin-1, or endomorphin-2, with [Leu^31^, Pro^34^]-NPY. Second, our studies were restricted to testing at 2 days after incision. In contrast, endogenous analgesic synergy was revealed with MOR- and Y1R-selective antagonists that were tested 21 days after incision. Thus, the question remains as to whether MOR and Y1R agonists interact in a supra-additive manner at later time points. To further test the idea that endogenous synergy predicts drug synergy, future studies could test the combined administration of MOR- and Y1R-selective agonists in models of chronic pain lasting 3 weeks or longer. Indeed, i.t. Y1R agonists effectively inhibit neuropathic hyperalgesia when tested weeks after injury (37, 54).

The molecular mechanisms underlying the endogenous MOR and Y1R synergy remain unknown, but several possible mechanisms exist. First, MOR and Y1R may form receptor–receptor interactions, such as the formation of heterodimers (17, 18). The formation of heterodimers or oligomerization between GPCR receptors can markedly potentiate signal transduction (55, 56). Second, MOR and Y1R may undergo signal transduction interactions. The assumption is that Y1R and MOR coexist on neurons and share a common pool of G proteins; therefore, activation of one receptor may cause redistribution of its G proteins and increase the sensitivity of the other receptor. For example, binding of an endogenous ligand, such as NPY to Y1R, may shift the affinity of endogenous ligand binding to the separate GPCR MOR (57). Additionally, both MOR and Y1R may synergistically work through downstream effectors. The free Gβγ released from the agonist-induced dissociation of both the MOR and Y1R G_i_ heterotrimers may coactivate protein kinase C (PKC), phospholipase C (34, 58), or PKC epsilon (28) to synergistically oppose LS. Third, peptide hormones like NPY can modulate neurotransmission by recruiting other GPCRs from the interior of the cell to the cell membrane (59). Coincident activation of Y1R and MOR may allow recruitment of MORs to the cell membrane and a sensitization of MOR signal transduction (59–61). Future experiments should further evaluate how Y1R and MOR interact mechanistically to promote endogenous synergy, for example after the i.t. administration of antibodies directed to NPY or enkephalin.

Our DRG- and spinal cord–conditional knockout data indicate that DH neurons mediate endogenous MOR::Y1R synergy. Interestingly, on the one hand, we found that BIBO3304 reinstated incision-induced mechanical hypersensitivity; on the other, we found that spinal *Npy1r* was not required for the resolution of mechanical sensitivity following incision. A possible explanation for these seemingly incongruent results is that *Npy1r^l^*^oxP/loxP^; *Lbx1*^Cre^ mice may exhibit enhanced compensatory spinal inhibitory GPCR signaling from other receptors (i.e. KOR, DOR MOR, etc.) to resolve hypersensitivity as a homeostatic consequence to the loss of *Npy1r*. Thus, to address this possibility, future studies could interrogate LS with an inducible Cre-line approach (for example, the development and use of a tamoxifen-inducible Cre line for spinal cord study, i.e. *Lbx1*^CreER^) and study *Npy1r* and/or *Oprm1* knockout after the resolution of injury. Indeed, we have successfully used a similar approach for temporally controlled NPY knockdown to reinstate injury-induced hypersensitivity (11).

Our results reveal *Npy1r* and *Oprm1* coexpression in the DH (Fig. 1). Indeed, previous immunohistochemical (62), neurophysiological (63), and transcriptomic (64–66) studies find evidence of colocalization of these two inhibitory GPCRs in DH neurons. Future studies may seek to further characterize the identity and function of these interneurons. A harmonized atlas of mouse single-cell RNA sequencing data results presents one particularly promising subpopulation that robustly coexpresses *Npy1r* and *Oprm1*, termed “Excit-8” neurons (64). Excit-8 neurons are glutamatergic, in the Reelin (*Reln*) family, and express gastrin-releasing peptide (*Grp*). Indeed, GRP interneurons robustly express *Npy1r* (54, 67) and exhibit functional responses to MOR agonists (68).

In summary, the current study establishes the existence of supra-additive endogenous MOR and Y1R signaling in the spinal cord DH that maintains LS in remission. Further, we provide a strong basis for future investigations of the mechanisms involved in MOR-Y1R endogenous synergistic signaling and the cellular subpopulations in the DH that drive LS.

## Materials and methods

### Animals

Adult C57Bl/6NCrl (Charles River, #027), *Npy1r*^loxP/loxP^ (courtesy of Herbert Herzog (69)), *Pirt*^Cre^ (courtesy of Xinzhong Dong (70)), and *Lbx1*^Cre^ (courtesy of Carmen Birchmeier (71)) mice were group housed, provided access to food and water ad libitum, and maintained on a 12:12 h light:dark cycle (lights on at 7:00 AM) in temperature and humidity controlled rooms. To generate conditional knockout mice, we first crossed *Pirt*^Cre^ or *Lbx1*^Cre^ mice to *Npy1r*^loxP/loxP^ mice and then bred males carrying both the Cre allele and the floxed allele with homozygous *Npy1r*^loxP/loxP^ female mice. We carefully looked for unexpected generation of the recombined allele by polymerase chain reaction as recommended (72). We detected evidence of unexpected recombination in DNA from tissue where female *Lbx1*^Cre^ mice were bred and did not use those mice for any experiments. For this reason, the above-described breeding strategy was used. Male and female mice were used in all experiments. Experiments were not powered to detect significant sex differences, but there were no trends pointing to any sex differences, and the data from both sexes were pooled. All experiments were carried out in accordance with guidelines from the International Association for the Study of Pain and with the approval of the Institutional Animal Care and Use Committees of the University of Pittsburgh.

### Drugs

**Table:** 

Chemical	Source	Dilutant information
BIBO 3304 trifluoroacetate	TOCRIS—Cat:2412	Diluted in a vehicle solution of ETOH, castor oil and saline in a 1:1:8 ratio
CTOP	TOCRIS—Cat:1578	Diluted in saline
PEAQX tetrasodium salt	TOCRIS—Cat:5018	Diluted in saline
[Leu^31^, Pro^34^]-NPY	TOCRIS—Cat:1176	Diluted in saline
DAMGO	Tocris—Cat:1171	Diluted In Saline

### Intrathecal injections

As previously described (11–13), mice were lightly restrained in a towel and a 30G ½ inch needle attached to a 25-μL Hamilton syringe was inserted into the subarachnoid space between the L5/L6 vertebrae at an angle of 30–45° to the horizontal plane. The needle was advanced until a reflexive tail flick was observed, at which time 5 μL of drug or vehicle was slowly administered. The needle was held in place for 10 s, withdrawn, and then the mouse was returned to its testing chamber.

### Plantar incision model

Postoperative hyperalgesia was induced by longitudinal incision of the plantaris muscle, as previously described (16, 73). Following antisepsis of the left hind paw with Chlorascrub and 70% ethanol, a #11 scalpel blade was used to make a 5-mm incision through the skin and fascia, beginning 2 mm from the proximal edge of the heel and extending toward the digits. The underlying muscle was raised with a curved forceps, extended 4 mm, and then incised longitudinally with the #11 scalpel blade, all while leaving the origin and insertion of the muscle intact. The overlying skin was closed with synthetic 5-0 sutures (PDS*II, Ethicon). Surgery was typically completed within 5–10 min. Surgeries were conducted under isoflurane anesthesia (5% induction followed by 1.5–2.0% maintenance). After suturing of the skin, triple antibiotic ointment (Neosporin, Johnson and Johnson) was applied to the surgical area. The sutures were removed 10 days after surgery.

### Behavioral testing

#### Mechanical hypersensitivity

Sensitivity to a nonnoxious mechanical stimulus was tested with an incremental series of 8 von Frey monofilaments of logarithmic stiffness (Stoelting, Wood Dale, IL, USA) that ranged in gram force from 0.008 to 6 g. The stimulation was applied lateral to the suture line. Filaments were applied to the skin with a slight bending of the filament for a maximum of 5 s. A clear withdrawal of the paw from the application of the stimulus was recorded as a positive response. The 50% withdrawal threshold was determined using the up-down method (74). Before commencement of each von Frey session, we acclimated the animals within individual Plexiglas boxes placed on the top of a stainless-steel mesh platform for 45 min.

#### Conditioned place aversion

A 2-day conditioning protocol using a biased chamber assignment was used for CPA. On the acclimation day (day 0), mice had free access to explore all chambers of a three-chamber conditioned place testing apparatus (side chambers: 170 × 150 mm; center chamber: 70 × 150 mm; height: 200 mm; San Diego Instruments) for 30 min. Mice were able to discriminate between chambers using visual (vertical versus horizontal black-and-white striped walls) and sensory (rough versus smooth textured floor) cues. For preconditioning (days 1 and 2), mice were again allowed to freely explore for 30 min during which their position was recorded via a 4 × 16 infra-red photobeam array and associated software (San Diego Instruments). For conditioning (days 3 and 4), each mouse's nonpreferred chamber was paired with the conditioning stimulus of a vehicle injection (i.t.), and the preferred chamber was paired with the conditioning stimulus of a BIBO3304:CTOP injection (10 ng, i.t.). Each morning, mice received an i.t. vehicle injection, were returned to their home cage for 5 min (to disassociate the injection with the chamber), and were then placed in the designated side chamber for 60 min. Six hours later, mice received BIBO3304:CTOP combo (10 ng, i.t.), were returned to their home cage for 5 min, and were placed into the BIBO3304:CTOP combo-designated chamber for 60 min. On test day (day 5), mice could freely explore all chambers and their position was recorded as during preconditioning for 30 min. Difference scores were calculated as the time spent in the chamber on test day minus the time spent in the same chamber during preconditioning.

#### Touch-evoked pERK

pERK was evoked by touch stimulation as previously described (13, 37). Twenty-one days after incision, mice received either i.t. injections of vehicle, BIBO3304 9.7 ng, CTOP 0.3 ng, or COMBO 10 ng (BIBO3304 9.7 ng + CTOP 0.3 ng). One hour later, mice were lightly anesthetized with isoflurane (1.5%), and the ventral surface of the ipsilateral hind paw was mechanically stimulated with a gentle 3-s stroke with a cotton swab from heel to toe. This was repeated every 5 s for 5 min. After an additional 5 min pause, mice were more deeply anesthetized with isoflurane and transcardially perfused with ice cold 0.01 M phosphate-buffered saline (PBS, Fischer Scientific), followed by 10% phosphate formalin buffer. Lumbar spinal cords were harvested and postfixed in the same fixative overnight at 4°C and then cryoprotected with 30% sucrose until total submersion (1–3 days).

#### Immunohistochemistry

Transverse spinal cord sections (30 μm) from L3 to L5 were cut on a sliding microtome (Leica, SM, 2000R). A series of sections, each 240 μm apart, were washed in 0.01 M PBS, blocked in 3% normal serum (goat; Gemini Bioproducts) containing 0.3% Triton X-100 (Sigma-Aldrich) in 0.01 M PBS for 1 h, and then incubated with primary rabbit antibody antiphosphorylated-ERK1/2 antiserum (1:1000, Cell Signaling) at 4°C for 24 h on a shaker. The following day, sections were again washed in 0.01 M PBS and incubated for 1 h at room temperature with the secondary conjugated antibody (1:1000, Invitrogen: goat antirabbit Alexa Fluor 488). The sections were washed in 0.01 M phosphate buffer, mounted, and coverslipped with VECTASHIELD HardSet Antifade Mounting Medium with DAPI. At least six good quality sections from segment L4 were selected from each subject for microscopy.

#### Fluorescence in situ hybridization (RNAscope)

Mice were transcardially perfused with ice cold 1× PBS followed by 10% buffered formalin and spinal cords, and DRGs were extracted via blunt dissection, postfixed in 10% formalin (2–4 h), and then placed in 30% sucrose at 4°C until the tissue sank (∼48–72 h). Twenty-micrometer-thick L3–L4 floating spinal cord sections were obtained on a vibrating microtome, and 12-μm thick L3–L4 DRGs were cut on a cryostat and mounted on Superfrost Plus Microscope slides and air dried overnight at room temperature. Slides underwent pretreatment for FISH consisting of 10 min xylene bath, 4 min 100% ethanol bath, and 2 min RNAscope H_2_O_2_ treatment. Next, the FISH protocol for RNAscope Fluorescent v2 Assay (Advanced Cell Diagnostics) was followed for hybridization to marker probes (Advanced Cell Diagnostics: Mm-Npy1r-C2, Cat No. 427021-C2, and Mm-Oprm1, Cat No. 315841). Slides were then coverslipped with VECTASHIELD HardSet Antifade Mounting Medium with DAPI.

#### Microscopy

All images were captured on a Nikon Eclipse Ti2 microscope using a 20× or 40× objective and analyzed using NIS-Elements Advanced Research software v5.02. An examiner blinded to treatment and sex counted the number of positive pERK cells in laminae I and II. Cells with at least three puncta associated with a DAPI nucleus were considered positive for FISH.

#### Synergistic interaction analysis

Drug interactions were evaluated using the open access software SiCoDEA (Single and Combined Drug Effect Analysis) (75). First, the percent maximum possible effect (%MPE) was calculated from the animal von Frey mechanical hypersensitivity data at the peak (60 min postdrug) time point as follows: %MPE = 100 × (postinjection threshold − preinjection threshold)/(postinjury threshold − preinjection threshold). The %MPE data for each animal with each dose combination was then analyzed with SiCoDEA. The parameters chosen for analyses were as follows: normalization method was “Max Signal,” inhibition/viability was “Viability,” and remove outlier was “0” for no outlier removal. Based on the curves and the calculated *R*^2^, the model that best fit our data was selected for analysis with the ZIP model. Specifically, for the endogenous synergy data (Fig. 2), the best model (and therefore with a higher *R*^2^) was the Log-logistic with two parameters (log-logistic[01]). For the exogenous synergy data (Fig. 5), the best model (and therefore with a higher *R*^2^) was the Log-logistic with four parameters (log-logistic). The resulting combination index (CI) matrix heat plot and values were then exported and compiled in Adobe Illustrator. The resulting CI matrix plots demonstrate two different representations of the same results: CI values and dots with dimensions proportional to the values of CI reported (the strength of synergy or antagonism). A CI value = 0 is additive, a value <0 is supra-additive (synergistic), and a value >0 is subadditive (antagonistic).

#### Statistical analyses

Data were analyzed by one- or two-way ANOVAs, followed by Dunnet, Tukey, and Holm–Sidak multiple comparison tests as appropriate. Data from dose–response curves were also analyzed as area under the curve. Nonlinear regression analyses were performed to fit curves for the data of %MPE. All statistical analyses were performed in GraphPad Prism 9.0 except combinatorial drug analysis (synergistic analysis) which was performed with SiCoDEA. Adobe Illustrator 2022 and Biorender.com were used to make the graphics. All data are presented as mean ± SEM. Statistical significance was determined as *P < 0.05.

## Data Availability

All data are included in the manuscript.

## Preprint

This manuscript was posted on a preprint server: https://www.biorxiv.org/content/10.1101/2022.07.05.498802v1.
